# A Comprehensive Insight and In Silico Analysis of CircRNAs in Hepatocellular Carcinoma: A Step toward ncRNA-Based Precision Medicine

**DOI:** 10.3390/cells13151245

**Published:** 2024-07-24

**Authors:** Rana A. Youness, Hossam A. Hassan, Tasneem Abaza, Ahmed A. Hady, Hekmat M. El Magdoub, Mohamed Ali, Johannes Vogel, Markus Thiersch, Max Gassmann, Nadia M. Hamdy, Mostafa A. Aboouf

**Affiliations:** 1 Molecular Genetics Research Team (MGRT), Molecular Biology and Biochemistry Department, Faculty of Biotechnology, German International University (GIU), Cairo 11835, Egypt; rana.youness21@gmail.com (R.A.Y.); hossam.ali.hassan19@gmail.com (H.A.H.); tasneem.abaza11@gmail.com (T.A.); 2 Biotechnology Program, Institute of Basic and Applied Sciences (BAS), Egypt-Japan University of Science and Technology (E-JUST), New Borg El-Arab City 21934, Egypt; 3 Clinical Oncology Department, Faculty of Medicine, Mansoura University, Mansoura 35511, Egypt; hadyonc@mans.edu.eg; 4 Biochemistry Department, Faculty of Pharmacy, Misr International University, Cairo 19648, Egypt; hekmat.magdoub@miuegypt.edu.eg; 5 Department of Obstetrics and Gynecology, University of Chicago, Chicago, IL 60637, USA; mohamed.ali@bsd.uchicago.edu; 6 Clinical Pharmacy Department, Faculty of Pharmacy, Ain Shams University, Cairo 11566, Egypt; 7 Zurich Center for Integrative Human Physiology and Institute of V. Physiology, University of Zurich, 8057 Zurich, Switzerland; jvogel@vetphys.uzh.ch (J.V.); markus.thiersch@uzh.ch (M.T.); maxg@access.uzh.ch (M.G.); 8 Biochemistry Department, Faculty of Pharmacy, Ain Shams University, Abassia, Cairo 11566, Egypt

**Keywords:** circRNAs, HCC, initiation, progression, metastasis, epigenetics, theranostics, ncRNA, in silico, bioinformatics

## Abstract

Circular RNAs (circRNAs) are cardinal players in numerous physiological and pathological processes. CircRNAs play dual roles as tumor suppressors and oncogenes in different oncological contexts, including hepatocellular carcinoma (HCC). Their roles significantly impact the disease at all stages, including initiation, development, progression, invasion, and metastasis, in addition to the response to treatment. In this review, we discuss the biogenesis and regulatory functional roles of circRNAs, as well as circRNA–protein–mRNA ternary complex formation, elucidating the intricate pathways tuned by circRNAs to modulate gene expression and cellular processes through a comprehensive literature search, in silico search, and bioinformatics analysis. With a particular focus on the interplay between circRNAs, epigenetics, and HCC pathology, the article sets the stage for further exploration of circRNAs as novel investigational theranostic agents in the dynamic realm of HCC.

## 1. Introduction

Circular RNAs (circRNAs) have recently been in the spotlight, yet their origins date back over four decades [1,2]. The exploration of circRNAs goes back to observations in murine retroviruses and plant pathogenic viruses, namely viroids. In 1979, the circular structure of circRNAs was confirmed through the electron microscope analysis of eukaryotic cells. Subsequently, in 1986, circRNAs were identified in the hepatitis delta virus, marking their initial detection in humans [1,3]. These early breakthroughs established the basis for elaborating on the diverse functions of circRNAs in biological processes [4,5,6,7,8].

## 2. CircRNA Nomenclature

The dynamic landscape of circRNA research and the lack of standardized nomenclature pose a major challenge [4,9,10]. Existing databases such as circBase 0.1 http://www.circbase.org/ [11] (accessed on 17 May 2023) use arbitrary numbering and have limited knowledge of the host gene and chromosomal location of some circRNAs. To solve this problem, a circRNA nomenclature system was developed based on the host gene and precise start/end positions within the hosting gene. This innovative approach was implemented in the newly developed Circbank Database, http://www.circbank.cn/ (accessed on 17 May 2023) which contains 140,790 human circRNAs. This database not only comprehensively organizes circRNA data but also provides valuable information, including microRNA (miRNA) binding sites, conservation, m6A modifications, circRNA mutations, protein-coding potential, and predicted internal ribosome entry sites (IRESs), providing a basis for further development of circRNA nomenclature and functions [12].

According to Bagheri Moghaddam et al. [13], the CircBank Database recently introduced a new nomenclature system for circRNAs based on the host genome and the specific location of the circRNA within that gene. Specifically, the upstream circRNA is assigned the starting number. Regarding intergenic circRNAs, the naming convention follows the format “hsa-circChrom#_#”, where the chromosomal number denotes the first number, and the second number follows the same rules as circRNAs derived from coding genes.

## 3. CircRNA Classification

The classification of circRNAs is based on their origin. The exonic circRNAs (EcircRNAs) originate from the coding regions of genes and play a crucial role in controlling genes [14] after they have been transcribed [14,15]. They act like conductors in a symphony, specifically by sequestering miRNAs [16,17]. In simpler terms, they function as a control center, fine-tuning the orchestra of genes in our cells after the transcription of genetic information [18]. In contrast, circular intronic RNAs (ciRNAs), originating from intronic regions of genes, are primarily localized within the nucleus, where they intricately coordinate transcriptional dynamics. Last but not least, a unique composition of exon–intron circRNAs (EIcircRNAs) are involved in complex interactions with RNA polymerase II, a key enzyme involved in the transcription of genes [10]. This interplay with RNA polymerase II underscores the multifaceted role of EIcircRNAs in modulating gene expression processes within the cellular nucleus [19].

CircRNAs play vital roles in physiology and pathology, acting as sponges for miRNA, regulating gene transcription, controlling RNA-binding proteins, and producing functional peptides [6,20]. Interestingly, almost 25% of eukaryotic genes that code for proteins in the mammalian brain are encoded by circRNAs. For example, circAcbd6 has a role in transforming neural stem cells into cholinergic neurons. This is achieved by inhibiting the function of miR-320-5p, thereby affecting the expression of Osbpl2, hence providing valuable insights into the mechanisms by which circRNAs promote or inhibit neurogenesis [21].

## 4. CircRNA Biogenesis

**Figure 1:**
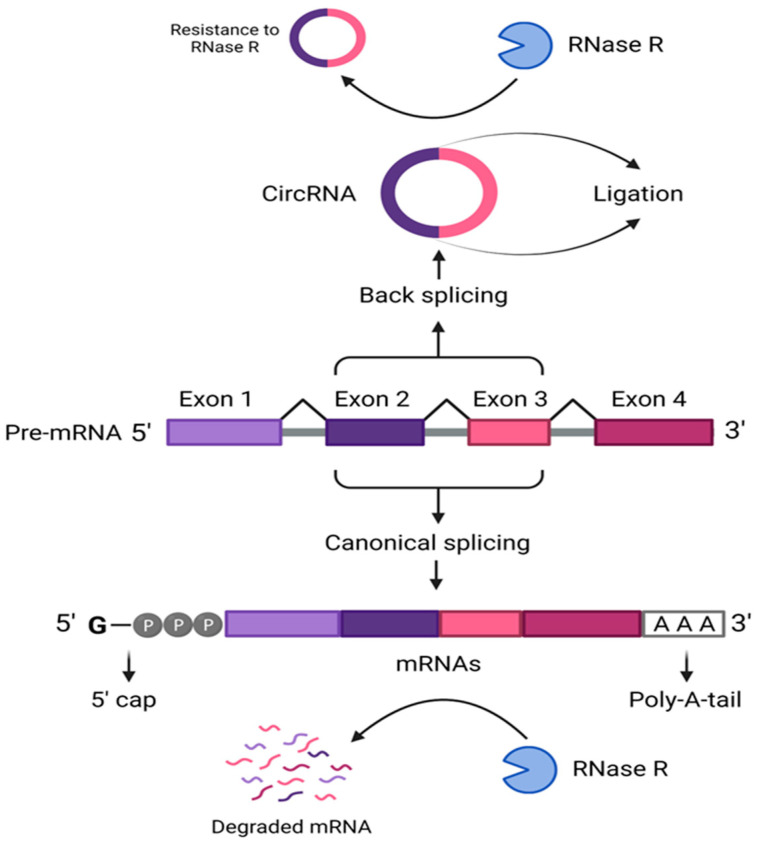
Splicing of circRNAs versus mRNAs. This figure demonstrates the non-canonical back splicing of the circRNAs from the pre-messenger RNA, showing its single-strand closed-loop structure, which is resistant to RNase, versus the canonical splicing of the mRNA with its polyadenylated 3′ tail. [CircRNA: circular RNA; mRNA: messenger RNA, Poly-A-tail: polyadenylated tail].

CircRNAs are endogenously synthesized from exons by the “Exons Back Splicing” method, a form of non-canonical messenger RNA splicing [5,22]. CircRNAs are distinguished by their single-strand closed structure, produced via ligating the 5′-3′-splice, and donor–acceptor sites [23,24,25]. This contrasts the normal splicing of the pre-mRNAs finished with a 5′ cap and 3′ polyadenylated tails, as shown in Figure 1 [26,27]. Difference points between circRNA and mRNA are summarized in Table 1, indicating the direction of the splicing, the pre-mRNA, the ligation sites, the structure, the parent genetic material, and susceptibility to RNase R [9,28].

## 5. Mechanisms of CircRNA Biogenesis

**Figure 2:**
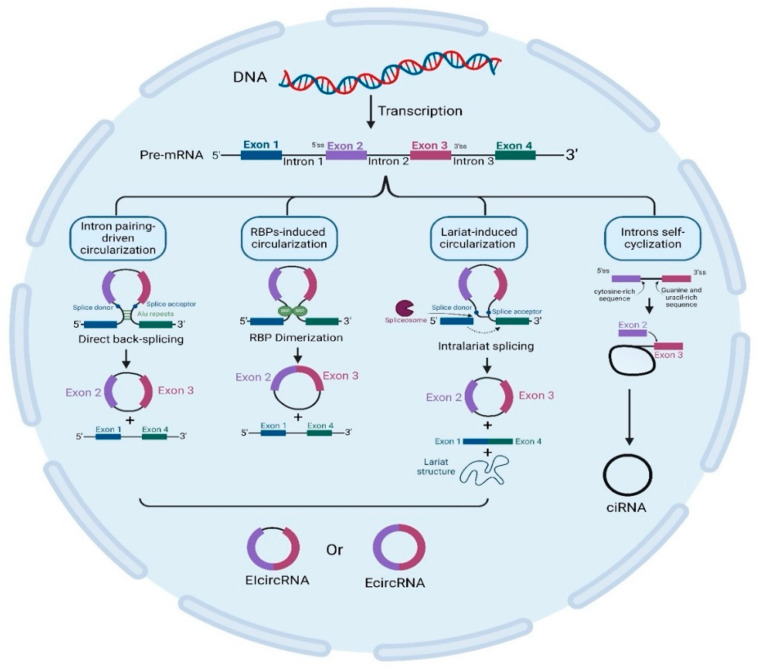
CircRNA biogenesis. This figure illustrates how circularization can be induced by intron pairing, RBPs, and lariat, which is triggered by spliceosomes and results in the synthesis of EIcircRNA or EcircRNA. It also depicts introns self-cyclization producing ciRNA. [CircRNA: circular RNA; ciRNA: intron circRNA; EIcircRNA: exon–intron circular RNA; Ecirc: exonic circular RNA; RBPs: RNA-binding proteins].

As mentioned earlier, circRNAs are sub-classified into three categories, EcircRNA, ciRNA, and EIcircRNA, as illustrated in Figure 2 [29,30]. CircRNAs are generated via various mechanisms. Table 2 demonstrates the different mechanisms involved in circRNA circularization, including intron pairing-driven circularization, RBP-induced circularization, lariat-induced circularization, and intro self-cyclization. Following their biogenesis, circRNAs are regulated by the associated miRNA levels in their producing cells and then transferred to body fluids through exosomes [28].

## 6. CircRNAs and Cancer Pathology

In the context of cancer pathology, circRNAs are critical players with significant implications across diverse cancer types, spanning from brain cancer to myeloma. The expression patterns of circRNAs exhibit associations with crucial stages in cancer progression, impacting immune response, cellular differentiation, pluripotency, apoptosis, and angiogenesis. Investigating the specific types of circRNAs and their precise chromosomal locations in distinct cancer types provides valuable insights into their roles and actions [41]. CircRNAs have been shown to possess significant implications in HCC development and advancement. They contribute to cell proliferation, tumor metastasis, evasion of immune responses, and resistance to drugs [42,43,44,45,46,47].

## 7. HCC Prevalence and Etiology

The most prevalent type of primary liver cancer and the third leading cause of cancer death globally is hepatocellular carcinoma (HCC). Regarding frequency, HCC ranks ninth in women and fifth in men. Its incidence rates vary across different regions worldwide [48,49,50,51]. According to Ferlay et al., one million individuals will be affected annually by HCC in one way or another by 2025 [52].

**Figure 3:**
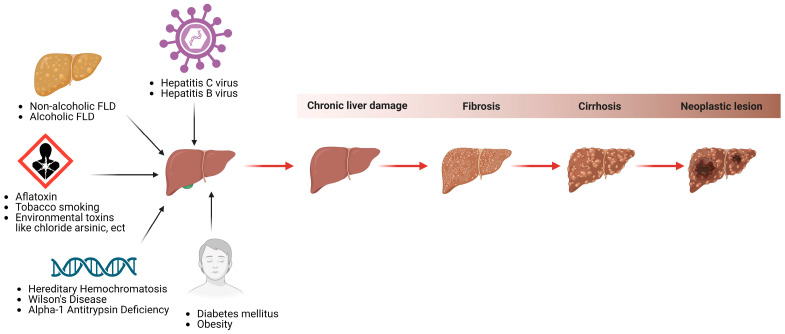
Some HCC predisposing factors. This figure depicts some hereditary diseases (hemochromatosis, Wilson’s, alpha-1 antitrypsin deficiency), acquired conditions (hepatitis C and B viruses, non-alcoholic FLD and alcoholic FLD), metabolic abnormalities (diabetes mellitus and obesity), and environmental risk factors (aflatoxins, tobacco, and others) that predispose people to chronic liver damage and hepatic cancers. [HCC: hepatocellular carcinoma, FLD: fatty liver disease].

The etiology of HCC is multifactorial, involving interactions between various causative agents [53,54,55,56]. HCC can result from long-term viral infections such as chronic hepatitis B virus (HBV) and hepatitis C virus (HCV) [57,58,59]. Metabolic problems like obesity and diabetes, especially in women [57,59,60,61,62,63], and inherited conditions like hemochromatosis and Wilson’s disease can also cause HCC, as shown in Figure 3. [52,64,65,66]. Biological and molecular mechanisms in HCC involve either tumor suppressor genes or oncogenes [51,67], interleukins [2,68,69], immunoglobulin-like receptors [70,71,72], and/or various cytokines and their polymorphisms [57,58,73].

Some hereditary conditions become more significant as we age, contributing to HCC risk. It is worth mentioning that around 10–20% of HCC cases occur in individuals without liver cirrhosis. Non-alcoholic fatty liver disease (NAFLD) represents an independent risk factor for HCC and is often linked to obesity due to the increased consumption of fatty diets [57,74]. Other risk factors include exposure to aflatoxins, excess iron in the body, and even smoking. Most of these risk factors promote the development of cirrhosis, which is present in more than 80–90% of HCC cases [75], as depicted in Figure 3.

The term poor prognosis usually accompanies HCC because HCC lacks symptoms in its early stages [76]. It is worth mentioning that survival rates are directly linked to HCC early diagnosis and, hence, a better prognosis. Another reason that may contribute to HCC’s bad prognosis is the fact that HCC represents the end stage of liver disease, so there is minimal reserving capacity at this stage [77]. Further, HCC itself, being an aggressive cancer that is highly metastasized, contributes as well to the disease’s poor prognosis [78]. Hence, even if HCC is appropriately diagnosed, it is still difficult to control [79].

## 8. HCC Molecular Heterogeneity

The underlying pathogenic condition(s) affect(s) the molecular pathways involved in the etiology of HCC. HCV-mediated hepatocarcinogenesis primarily occurs through host–viral protein interactions, particularly involving the core, non-structural proteins NS3, NS4A, and NS5A [80,81].

Abundant and enduring RNA molecules, such as miRNAs or long non-coding RNAs (lncRNAs), showcase a manifold of abilities in physiological and pathological contexts. They function as repositories, regulatory elements, catalysts of translation, identifiers, and healthy tumor suppressors. Hence, their significance was demonstrated across multiple cancer types like colon, liver, and breast [82]. LncRNAs can regulate gene expression in three ways: epigenetic, transcriptional, and post-transcriptional [83,84]. LncRNAs have been well investigated in terms of their role in the regulation of cancer. For example, in HCV, lncRNA-ATB is highly associated with fibrosis and may also be involved in developing HCC [4,85,86,87,88,89,90,91,92]. In this aspect, lncRNA-ATB was reported to promote tumor metastasis via the induction of epithelial–mesenchymal transition (EMT) [93]. On the other hand, miRNAs function as post-transcriptional regulators by binding mRNA and inhibiting its translation into a protein. Several studies have identified an association between dysregulated miRs and the development of HCC [94,95].

## 9. Role of CircRNAs in HCC

The functions of circRNAs in HCC are complicated, as they can act either as good or evil, being tumor suppressors or oncogenes, respectively [96,97,98,99]. CircRNAs play various functional roles in tuning the initiation, development, progression, and metastasis of HCC.

## 10. CircRNAs Act as miRNA Sponges or Decoys

**Figure 4:**
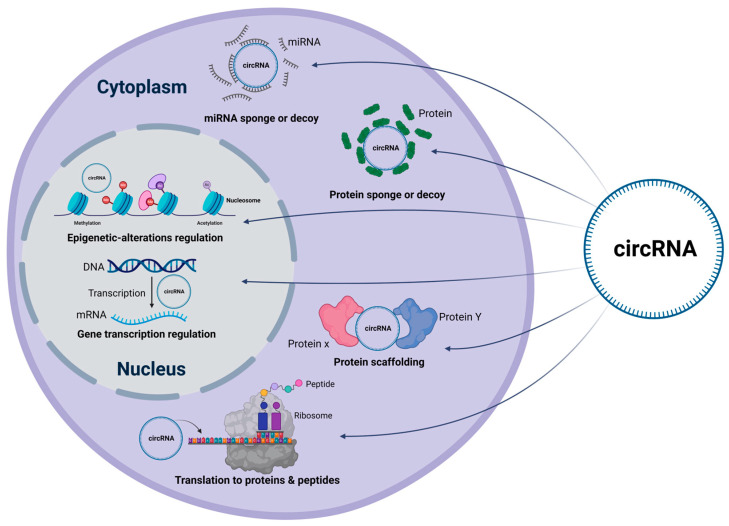
Regulatory mechanisms of circRNAsin HCC. This figure summarizes the different mechanisms by which circRNAs contribute to HCC. CircRNAs function as miRs or protein sponges. They can also scaffold cellular proteins and regulate the latter’s translation in the cytoplasm. Further, circRNAs can alter epigenetic regulations and modulate gene transcription within the nucleus. [CircRNA: circular RNA; miRNA: microRNA; mRNA: messenger RNA].

CircTRIM33-12 sponging miR-191 upregulates the expression of tet methylcytosine dioxygenase 1 (TET1), which lowers the levels of 5-hydroxymethylcytosine in HCC cells [100]. By acting as a decoy for miR-9, CircMTO1 inhibits cell proliferation in HCC and functions as a tumor suppressor by upregulating p21 [101]. Similarly, circHIPK3 promotes HCC proliferation by sponging some miRs, including miR-124 [97] and miR-29b [102], as illustrated in Figure 4, leading to the release of their target genes responsible for cell growth regulation. Other studies demonstrate the oncogenic behavior of HIPK3 through sponging miR-338-3p [103]. By sponging miR-3619-5p, increasing catenin beta 1 (CTNNB1) expression, and triggering Wnt/β-catenin signaling, CircZFR controls cell proliferation, the epithelial–mesenchymal transition, and the Wnt/β-catenin pathway [104]. Another circRNA, circFBLIM1, acts as a competing endogenous RNA (ceRNA) that enhances HCC progression via sponging miR-346 [105]. CircMAT2B promotes glycolysis and HCC malignancy by sponging miR-338-3p to activate the pyruvate kinase M2 (PKM2) axis under hypoxia [43]. CircTP63 increases ZBTB18 expression by sponging miR-155-5p, which advances HCC. The latter was reported to positively correlate with mortality rates in HCC patients [106]. However, by upregulating tissue inhibitor of metalloproteinase 3 (TIMP3), a well-known tumor suppressor that functions by sponging miR-17-3p and miR-181b-5p, hsa_circ_0001445 (cSMARCA5) suppresses the migration and proliferation of HCC cells [46].

A recent study proposed that circ_0001806 expedites HCC advancement by upregulating matrix metalloproteinase (MMP)-16 expression by inhibiting miR-193a-5p [107]. Another preliminary investigation, yet to be approved, revealed that circYTHDF3 fosters liver carcinogenesis via the miR-136-5p/chromobox 4 (CBX4)/vascular endothelial growth factor (VEGF) pathway [108]. Furthermore, earlier research reveals that circCFH stimulates HCC by modulating cellular functions via the miRNA 377-3p/RNF38 axis, including proliferation, apoptosis, migration, invasion, and glycolysis [109]. Lastly, circRNA CDR1as affects HCC progression by interacting with markers and miR-1287 bands within the Raf1 pathways [110].

In patients with HCC, Hsa_circ_0085616 (circASAP1) induced pulmonary metastases by stimulating the proliferation of cells, in vitro colony formation, migration, and invasion [42]. CircASAP1 was reported to operate as a ceRNA for the endogenous colony-stimulating factor (CSF) and mitogen-activated protein kinase (MAPK) suppressors miR-326 and miR-532-5p. MAPK and CSF are known to mediate tumor-associated macrophage infiltration, which is also linked to cell invasion and proliferation [42].

## 11. CircRNAs Function as Protein Sponges or Decoys

CircBACH1 interacts with human antigen R (HuR), an RBP, leading to the downregulation of p27 expression, as shown in Figure 4. Through the interferon-responsive sequence motif in the p27 5′-untranslated region, this interaction prevents translation. HuR transport and accumulation in the cytoplasm are similarly facilitated by CircBACH1 [111]. By competitively binding to fragile X mental retardation protein (FMRP), on the other hand, CircZKSCAN1 functions as a tumor suppressor by influencing the translation of cell division cycle and apoptosis regulator protein 1 (CCAR1) mRNA and blocking the Wnt signaling pathway (Table 3) [112].

## 12. CircRNAs Can also Serve as Scaffolding for Proteins

CircAMOTL1 facilitates the translocation of c-myc, 3-phosphoinositide-dependent kinase 1 (PDK1), AKT serine/threonine kinase 1 (AKT1), and signal transducer and activator of transcription 3 (STAT3) to the nucleus. Their target genes’ expression is modulated by this activity [113,114,115]. NR2F6 transcription and the advancement of HCC are triggered by CircRHOT1, which recruits TIP60 to the nuclear receptor subfamily 2 group F member (6NR2F6) promoter [116]. Another downregulated circRNA, hsa_circ_0020007 (circADD3), has been linked to vascular invasion and distant and intrahepatic metastasis of HCC, as summarized in Table 3. Mechanistically, circADD3 promotes the ubiquitination of EZH2 and the subsequent degradation of that protein. CircADD3 boosts the interaction between EZH2 and cyclin-dependent kinase 1 (CDK1) to accomplish this activity. The expression of several anti-metastatic genes, including dampening circADD3 itself, is increased when EZH2 is downregulated. This is achieved by lowering the histone tri-methylation marker H3K27me3 on the promoter regions of the anti-metastatic genes [117].

In HCC, CircPABPC1, another circRNA, directly feeds ITGβ1 to the proteasome for ubiquitin-independent degradation, demonstrating tumor-suppressive activity [118].

CircRNAs are also involved in regulating gene transcription. CircIPO11, for example, binds topoisomerase I (TOP1), which triggers GLI family zinc finger 1 (GLI1) transcription. This interaction leads to activating the Hedgehog signaling pathway [119]. It has also been demonstrated that circRNAs play a role in translating proteins or peptides. circCTNNB1 produces a new 370-amino-acid β-catenin isoform. This isoform is generated through the circularization process, which leads to translation termination at a new stop codon. This mechanism promotes HCC cell growth through the Wnt signaling pathway [120].

Last but not least, circRNAs were shown to regulate epigenetic alterations. For example, circSOD2 induces an epigenetic alteration that drives HCC progression by activating the JAK2/STAT3 signaling pathway [121]. In summary, Table 3 classifies the different circRNAs involved in HCC according to their functional roles while describing the corresponding mechanisms.

## 13. CircRNA–Protein–mRNA Ternary Complexes

Ternary circRNA–protein–mRNA complexes play crucial roles in regulating mRNA stability and translation. These complexes involve circRNA interactions with RBPs and mRNAs simultaneously. For instance, circNSUN2 can assemble into a complex with high mobility group A (HMGA2) mRNA and insulin-like growth factor 2 mRNA-binding protein 2 (IGF2BP2) to stabilize mRNA, triggering EMT and enhancing the aggressiveness of colorectal cancer (CRC) [122].

Moreover, circPOK functions differently than its linear counterpart, Pokemon, a tumor suppressor gene. CircPOK promotes the stability of VEGF mRNA and interleukin 6 (IL6) via interacting with the interleukin enhancer binding factor 2/3 (ILF2/3) complex. Additionally, it strengthens ILF2/3’s binding to the IL6 promoter. CircPOK regulates the tumor cell secretome both transcriptionally and post-transcriptionally [123]. Similarly, the circFNDC3B-IGF2BP3-CD44 mRNA ternary complex supports CD44 overexpression and mRNA stability [124].

In contrast, some circRNAs act as brakes in translation. With the aid of 11 complementary nucleotides and IRES, a tumor suppressor mRNA, a three-part complex is formed between circMALAT1 with paired box 5 (PAX5) and ribosome, causing mRNA breakdown. It additionally initiates the JAK2/STAT3 signaling pathway and functions as a sponge for miR-6887-3p [125].

Another circRNA was discovered to interfere with the translation initiation process. CircYap, which is known to interact with Yap mRNA, was found to interact with poly(A)-binding protein (PABP) and eukaryotic initiation factor 4 gamma (eIF4G), which bind to the 3′-tail and 5′-cap of the mRNA, respectively. This complex prohibits PABP and eIF4G interaction, thereby hindering Yap translation initiation [126].

CircRNAs play a role in the EMT, which is linked to drug resistance in HCC. Clear examples of dysregulated circRNAs involved in HCC drug resistance include higher expression of CircFoxo3 in adriamycin-resistant tissues, potentially contributing to resistance through the miR-199a-5p/ABCC1 pathway [127]. Furthermore, HCC cells’ release of CircUHRF1 wears down natural killer cells and increases their resistance to anti-programmed cell death protein 1 (PD1) immunotherapy [47]. On the other hand, reduced levels of circ_0003418 promote cisplatin resistance along with activation of the Wnt/β-catenin signaling cascade [128,129].

To sum up, circRNAs are viewed as regulatory ncRNA molecules that exert their effect directly by regulating the transcription and splicing of genes or indirectly by altering other regulators, including proteins and miRNAs (Table 3 and Figure 1). Accordingly, it is clear that the regulatory role circRNAs play in HCC remains a topic of ongoing research and needs further investigation.

## 14. Are circRNAs Involved in Therapeutic Resistance Experienced by HCC Patients?

In the context of resistance, circRNAs act as molecular sponges, preventing specific miRNA inhibitory effects on critical genes linked to resistance, which result in a loss of control [130,131]. So, surprisingly, the answer to the question is yes. CircRNAs influence crucial signaling pathways, affecting how HCC cells respond to therapeutic agents [132]. Their modulation extends to apoptosis and cell survival pathways, strengthening HCC cells against treatment-induced cell death and promoting resistance. Interestingly, interactions with RNA-binding proteins add a level of complexity to cellular responses following therapeutic interventions [133]. Sorafenib-induced resistance arose from circSORE that competitively activated the Wnt/β-catenin pathway by sponging miR-103a-2-5p and miR-660-3p [45]. Another proposed mechanism by which circSORE promotes sorafenib resistance involves binding to the oncogene Y-box binding protein 1 (YBX1). By blocking YBX1’s nuclear connection with the E3 ubiquitin ligase PRP19, this association inhibits the enzyme’s breakdown and increases the resistance to sorafenib [44].

## 15. Could circRNAs Act as Theranostic Agents for HCC Patients?

As mentioned earlier, HCC diagnosis is challenging. The main reason is the lack of exclusive, specific biomarkers for HCC. Moreover, there are not enough appropriate blood molecular markers for surveillance and early HCC diagnosis. The current biomarkers have low sensitivity and inconsistent specificity despite having different cut-off values [134].

Biomarkers like alpha-fetoprotein (AFP) are elevated in HCC and other pathological conditions such as chronic liver diseases. Further, it has also been demonstrated that around 40% of HCC patients present with normal AFP levels [135,136]. Des-γ-carboxy-prothrombin (DCP) has been studied as a promising biomarker for HCC [137]. Research is still ongoing in this area, and whole genome-wide sequences and DNA microarray analysis have identified markers of early HCC [138,139] that still need to be validated.

The concept of theranostics is linked to having a personalized health compass that guides treatment decisions and illuminates the unique individual molecular landscape [140]. This dynamic approach merges therapy and diagnostics, ensuring that medical interventions are tailored to the patient’s needs. In HCC, circRNAs act as molecular sensors, possessing traits ideal for early cancer detection and identifying subtle clues in tissues and body fluids [141,142]. These circRNAs, while still in the early stages of their therapeutic paths, have shown promising potential in preclinical trials [142]. For instance, in experiments involving circRNAs like circMYLK and circMAST1, the introduction of small interfering RNA (siRNA) demonstrated an ability to suppress tumor formation [141]. This is a precision strike against cancer cells guided by these circRNA navigators. Moreover, developing a plasma circRNA panel for diagnosing HCC is akin to having a sophisticated diagnostic tool, providing accuracy surpassing traditional markers. In this narrative of theranostics, circRNA emerges as a molecular marker and an active player, potentially transforming how we approach the personalized treatment landscape in HCC and beyond.

## 16. Exosomal circRNA Is a New Hot Area of Research

Exosomal hsa_circ_0051443, frequently downregulated in HCC cells, has been demonstrated to have anti-proliferative and pro-apoptotic properties in cells. This is facilitated by upregulating BRI1-associated kinase 1 as a consequence of miR-331-3p binding [143]. Exo_circ_79050 (also named hsa_circ_0009024) is a circRNA that is exosomal, originating from a “pseudogene” being upregulated in HCC as retrieved in silico from exoRBase v2.0 [144] (http://www.exorbase.org/exoRBaseV2/detail/detailInfo?id=exo_circ_79050&kind=circRNA&tab=profile, accessed on 18 May 2023). Its genomic position is chrY:19587210-19587507, with the positive strand upregulated in HCC. Moreover, four protein-coding exosomal circRNAs are upregulated in HCC, as shown in Table 4 (retrieved from exoRBase v2.0, http://www.exorbase.org/exoRBaseV2/browse/toIndex?kind=circRNA, accessed on 18 May 2023).

## 17. CircRNAs in HCC: Bioinformatics Analysis

**Figure 5:**
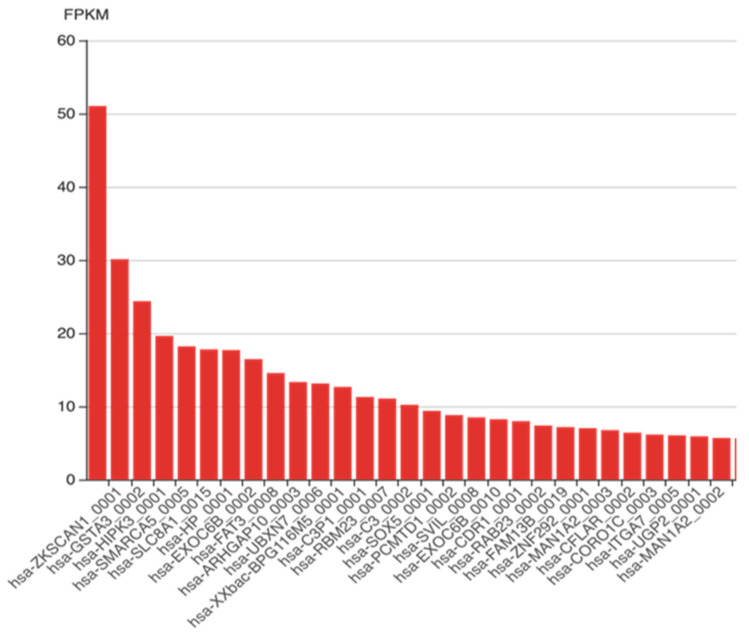
Liver-specific high expression of circRNAs. Figure 5 lists the most prevalent circRNA in the human liver in descending order of highest expression, as retrieved from circATlas 2.0, http://circatlas.biols.ac.cn/, accessed on 17 May 2023. [FPKM: fragments per kilobase of transcript per million mapped reads].

We accessed circAtlas 2.0 (http://circatlas.biols.ac.cn/) on 17 May 2023, for the liver’s most highly expressed human circRNAs, as shown in Figure 5.

## 18. CircRNAs in Different Liver Diseases

The upcoming chapter demonstrates the association between circRNAs and liver disease. In particular, the upregulated or downregulated circRNA expression patterns, circRNA-associated genes, sponged miRNAs, biological functions (e.g., proliferation, migration, and invasion), and molecular mechanisms (e.g., ceRNA, PI3K-AKT, FOXO, SIRT1, PPAR-a signaling pathways) of circRNA in various liver diseases are discussed. Data in Table 5 are retrieved from the circRNADisease v2.0 database (last updated January 2023 [145]), http://cgga.org.cn:9091/circRNADisease/, accessed on 17 May 2023.

Several circRNAs are downregulated in various liver diseases, each with its own specific biological function and molecular mechanism. In hepatocellular steatosis, circRNA_0046366 is downregulated and is associated with the miR-34a/PPAR-a signaling pathway, although the exact mechanism remains unknown. For liver fibrosis, hsa_circ_0070963, hsa_circ_0061893, and hsa_circ_0013255 are also downregulated. CircRNA_100395 in liver cancer suppresses growth and triggers apoptosis, potentially via controlling miR-1228. The JAK2/STAT5 pathway links the downregulation of CircScd1 in non-alcoholic fatty liver disease (NAFLD) to the advancement of fatty liver disease. In liver cancer, CircCDK13 inhibits the JAK/STAT and PI3K/Akt signaling pathways to prevent the disease from progressing. CircRNA_101764 is downregulated and linked to hsa-miR-181 in HBV-related HCC. Although their precise functions are uncertain, circ_03848, circ_08236, circ_13398, and circ_15013 are downregulated in liver regeneration. CircRNA-4099 in hepatitis triggers the keap1/Nrf2 and p38MAPK pathways and is associated with the aggravation of H2O2-induced injury through the regulation of miR-706, although the specific mechanism remains unclear.

On the other hand, several circRNAs are upregulated in various hepatic diseases and biological functions. In hepatotoxicity, hsa_circRNA_0000657, hsa_circRNA_0000659, hsa_circRNA_0003247, and hsa_circRNA_0001535 are upregulated, although the specific mechanism and associated miRNAs are not yet identified. For liver fibrosis, hsa_circ_0072765, hsa_circ_0071410, and hsa_circ_0054345 are upregulated, but their mechanisms remain unknown. In liver cancer, circZFR, circFUT8, and circIPO11 are also upregulated. CircMEG3 is also upregulated and has been shown to inhibit telomerase activity, shortening telomere lifespan and reducing Cbf5. Through the miR-155/FOXO3a pathway, circRNA-0067835 stimulates cell division and suppresses apoptosis in liver fibrosis. Circ_0091579 in liver cancer promotes proliferation and metastasis via miR-490-3p. For other hepatic diseases, hsa_circ_0003056 and hsa_circ_0067127 are upregulated in cancer, while mmu_circRNA_005186 is upregulated in ischemia/reperfusion injury, acting through the miR-124-3p/Epha2 pathway. CircRNA-1984 in hepatic stellate cells (HSCs) is related to fibrosis, possibly through the miR-146b pathway. Circ_0015756 and hsa_circ_0000594 are upregulated in hepatoblastoma, with hsa_circ_0000594 potentially acting through the mir-217/SIRT1 regulatory axis. CircFBLIM1 in hepatoblastoma promotes cell viability, proliferation, and invasion through the miR-346-ceRNA mechanism. CircHMGCS1 in hepatoblastoma regulates proliferation, apoptosis, and glutaminolysis, possibly through the miR-503-5p/IGF/PI3K/AKT axis and by regulating IGF2 and IGF1R expression. Circ-PWWP2A is upregulated in fibrogenesis and acts downstream of TGF-ß and LPS, possibly through the miR-203 and miR-223 pathways.

## 19. Expert Authors’ Opinions, Recommendations, and Future Perspective

To our knowledge, there are no clinical reports of circRNAs having a positive or negative impact on HCC by modifying an individual’s (epi)genes or polymorphism(s). CircRNAs have been linked to liver metastasis from CRC and HBV-mediated HCC [104,146]. Restrictive limitations on applying circRNAs as molecular markers in the HCC clinical field are related to inadequate clinical information about circRNA-potential axes and various HCC hallmarks.

Nevertheless, several known hsa-circRNA-miR downstream signaling targets were found, analyzed, and validated for HCC and/or liver disorders. To demonstrate their efficacy, these targets could be further investigated for other cancer types, such as BC or neurodegenerative diseases (NDDs).

Developing ncRNA precision therapeutic regimens can be achieved by targeting the hsa-circRNA-miR downstream signaling cascades through drug repurposing using molecular docking, followed by experimental validation of the selected drug’s efficacy.

## 20. Conclusions

Utilizing in silico databases, bioinformatics analysis (Supplementary File), and literature exploration, we emphasized in the current review the link between circRNAs and liver illnesses, particularly HCC. The significance of circRNAs as one of the epigenetic ncRNAs was highlighted. We compiled comprehensive background information regarding circRNA-related liver diseases with a particular emphasis on HCC. Specifically, we discussed the biological roles of circRNA in liver disorders, the molecular mechanisms by which they contribute to HCC as cancer molecular markers, the miRs they target to sponge, and the ultimate downstream signaling cascade. Nonetheless, the authors have shed light on a promising clinical implementation for circRNAs: their suitability as theranostic agents for HCC and their involvement in chemotherapeutic resistance experienced by some HCC patients. Yet these areas still need further investigation by the scientific community. Notably, circRNAs serve as promising targets for therapeutic interventions. CircRNAs are expected to have a novel function in tumor immunotherapy and/or controlling the tumor immune microenvironment in HCC. Ultimately, circRNAs have the potential to serve as an effective molecular tool in combating multi-drug resistance (MDR).

While emerging evidence suggests that circRNAs may play important roles in the pathogenesis and progression of HCC, it is important to note that the definitive establishment of their crucial role requires further investigation. Studies have shown that circRNAs are differentially expressed in HCC tissues, may regulate oncogenes and tumor suppressors, and can impact cellular processes such as proliferation, apoptosis, and metastasis. However, additional research is needed to fully elucidate their significance and mechanisms in HCC.

## Figures and Tables

**Table 1:** Differences between circRNA and mRNA during and after the RNA transcript maturation process.

Difference	CircRNA	Linear mRNA
Splicing	Back	Normal
Pre-mRNA	Non-canonical	Canonical pre-mRNA
Production	By ligation	With a free 5′-cap and 3′-tail
Structure	No free cap and tail	With a free cap and tail
Final structure	Covalent closed-loop structure; circular	Linear
Formed from	Exons located in the cytoplasm or the nucleus increase nuclear protein retention, and circRNAs within introns remain in the nucleus	Pre-mRNA from a DNA template in the cell nucleus
Resistant to RNase R	Yes	No

**Table 2:** Molecular circularization of circRNAs.

CircRNA Biogenesis	CircRNA Product	Biogenesis Mechanism	Refs.
Intron pairing-driven circularization	EcircRNAs or ElciRNAs	The method by which EcircRNA and EIcircRNA cyclize is known as “direct back splicing” or intron pairing-driven cyclization; pre-mRNA containing ALU repeats is sheared to form EcircRNA following reverse-base complementary pairing. EIciRNAs are produced if introns are kept in between exons.	[19,31]
RBP-induced circularization		RBPs (trans-acting factors) are Quaking, Muscleblind, and Fused-in Sarcoma. Circularization is facilitated by bridging comparable intronic regions. RBP dimerization links the 3′ and 5′ ends of circularized exons.	[32,33]
Lariat-induced circularization driven by spliceosomes		Exon circularization is spliceosome-dependent and is collected at the back-splicing site to help join the 5′-3′ donor–acceptor sites. Within lariat, internal splicing releases EcircRNAs or EIcircRNAs.	[34,35,36,37]
Intron self-cyclization	ciRNA	Intron self-cyclization is brought about by the 7 nucleotides of the G/U-rich sequence located near 1 exon and the 11 nucleotides of the C-rich sequence located near another exon in pre-mRNA. Three distinct kinds of circRNAs are produced: ciRNAs, EIcircRNAs, and EcircRNAs. A closed RNA loop (covalently EcircRNA) is formed when the 3′ end of an exon (5’ss) is joined to the 5′ end of either the same exon (single-exon circRNA) or an upstream exon (multiple-exon circRNA).	[22,38,39,40]

[CircRNA: circular RNA; ciRNA: intronic circRNA; EIcircRNA: exon–intron circular RNA; Ecirc: exonic circular RNA; RBP: RNA-binding protein].

**Table 3:** List of circRNAs, their functional roles, and mechanisms of action in HCC.

Functional Role	CircRNAs	Mechanism	Refs.
MiR sponge or decoy	CircTRIM33-12	increases the production of TET1 by the sponging of miR-191, lowering the levels of 5-hydroxymethylcytosine in HCC cells	[100]
	CircMTO1	downregulates p21 level by sponging oncogenic miR-9 to inhibit HCC progression.	[101]
	CircHIPK3	regulates AQP3 expression, sponges miR-124, alters cell proliferation and HCC migration	[97]
	CircZFR	regulates cell proliferation, epithelial–mesenchymal transition, Wnt/β-catenin via quenching miR-3619-5p, enhancing CTNNB1 expression and activating Wnt/β-catenin signaling	[104]
	CircFBLIM1	ceRNA that enhances HCC progression via sponging miR-346	[105]
	CircMAT2B	encourages HCC malignancy, glycolysis, and miR-338-3p quenching to activate the PKM2 axis under hypoxic conditions	[43]
	CircTP63	sponges miR-155-5p and thus increases ZBTB18 expression, which is positively correlated with mortality rates in HCC patients	[106]
	CircSMARCA5	TIMP3 expression via sponging miR-17-3p and miR-181b-5p	[46]
	Circ_0001806	expedites HCC advancement by upregulating MMP-16 expression through the inhibition of miR-193a-5p	[107]
	CircYTHDF3	fosters HCC via miR-136-5p/CBX4/VEGF pathway	[108]
	CircCFH	promotes HCC by influencing cellular proliferation, apoptosis, migration, invasion and glycolysis via miRNA 377-3p/RNF38 axis	[109]
	CDR1as	interacts with markers and miR-1287 bands within the Raf1 pathways to modulate HCC progression	[110]
	CircASAP1	ceRNA for miR-326 and miR-532-5p regulates the expression of MAPK1 and CSF-1 targets, facilitating invasion, HCC cell proliferation and infiltration of tumor-associated macrophages	[42]
	CircSORE	induces sorafenib resistance by competitively activating the Wnt/β-catenin pathway through miR-103a-2-5p and miR-660-3p	[45]
Protein sponge or decoy	CircBACH1	interacts with HuR; RBP downregulates p27 expression, blocks translation in the p27 5′-untranslated region by an interferon-responsive sequence element, encourages HuR translocation and cytoplasmic accumulation	[111]
	CircZKSCAN1	competitively binding FMRP to modulate the translation of CCAR1 mRNA and inhibiting the Wnt signaling pathway	[112]
Protein scaffold	CircAMOTL1	combines with c-myc, STAT3, PDK1, and AKT1 to promote their translocation to the nucleus, modulating the expression of their target genes.	[113,114,115]
	CircRHOT1	recruits TIP60 to NR2F6, initiating NR2F6 transcription and HCC progression	[116]
	CircADD3	protein scaffold inhibits HCC metastasis via CDK1-mediated EZH2 ubiquitination	[117]
	CircPABPC1	a tumor suppressor, directly delivering ITGβ1 to the proteasome for HCC ubiquitin-independent destruction	[118]
	CircSORE	causes sorafenib resistance by binding oncogenic YBX1 and blocking its nuclear interaction with E3 ubiquitin ligase PRP19	[44]
Gene transcription regulation	CircIPO11	binds TOP1 to trigger GLI1 transcription, with Hedgehog signaling activation.	[119]
Translation to proteins and peptides	CircCTNNB1	creates 370 amino acid β-catenin isoform, uses circularization to block translation at a new stop codon, uses Wnt to stimulate HCC cell development	[120]
Epigenetic alterations’ regulation	CircSOD2	induces epigenetic alteration to drive HCC progression by activating JAK2/STAT3 signaling.	[121]

[AKT1: AKT serine/threonine kinase 1; AQP3: Aquaporin 3; CBX4: chromobox 4; CCAR1: cell division cycle and apoptosis regulator protein 1; CDK1: cyclin-dependent kinase 1; CTNNB1: catenin beta 1; CSF: colony-stimulating factor 1; EZH2: enhancer of zeste homolog 2; FMRP: fragile X mental retardation protein; GLI1: GLI family zinc finger 1; HCC: hepatocellular carcinoma; ITGβ1: integrin β1; MAPK: mitogen-activated protein kinase; miR: microRNA; Hur: human antigen R; JAK2: Janus kinase 2; MMP: matrix metalloproteinase; NR2F6: nuclear receptor subfamily 2 group F member 6; PDK1: 3-phosphoinositide-dependent kinase 1; PKM2: pyruvate kinase M2; RBP: RNA-binding protein; STAT3: signal transducer and activator of transcription 3; TIMP3: metalloproteinase 3; VEGF: vascular endothelial growth factor; YBX1: Y-box binding protein 1.]

**Table 4:** Exosomal circRNAs upregulated in HCC urine or blood samples retrieved from exoRBase v2.0.

circID	circBase ID	Genomic Position	Strand	Gene Symbol
exo_circ_11335	NA	chr12:94169153-94186473	+	PLXNC1
exo_circ_23574	hsa_circ_0041462	chr17:3814322-3816270	−	NCBP3
exo_circ_71780	hsa_circ_0006320	chr8:22474954-22498112	+	PPP3CC
exo_circ_79066	hsa_circ_0001953	chrY:2953909-2961646	+	ZFY

http://www.exorbase.org/exoRBaseV2/browse/toIndex?kind=circRNA, accessed on 18 May 2023.

**Table 5:** CircRNAs in different liver diseases retrieved from circRNADisease v2.0 bioinformatics database search.

**Downregulated circRNAs**			
**CircRNAs**	**Hepatic Disease**/ **Biological Function**	**Mechanism**	**Molecular Mechanism/Associated miR (Sponged miR)**
circRNA_0046366	Hepatocellular steatosis	-	circRNA_0046366/miR-34a/PPAR-a signaling
hsa_circ_0070963, hsa_circ_0061893 and hsa_circ_0013255	Liver fibrosis	-	-
circRNAs_100395	Liver cancer	inhibits cell proliferation, induces apoptosis	miR-1228
circScd1	NAFLD	encourages the JAK2/STAT5 pathway, which causes fatty liver disease	-
circCDK13	Liver cancer	suppresses progression via JAK/STAT and PI3K/Akt signaling	-
circRNA_101764	HBV-related HCC	-	hsa-miR-181
circ_03848, circ_08236, circ_13398 and circ_15013	Liver regeneration	-	-
circRNA-4099	Hepatitis	unknown/triggers keap1/Nrf2 and p38MAPK	miR-706 aggravating H_2_O_2_-induced injury
**Upregulated circRNAs**			
**CircRNAs**	**Hepatic Disease**/ **Biological Function**	**Mechanism**	**Molecular Mechanism**/ **Associated miR (Sponged miR)**
hsa_circRNA_0000657, hsa_circRNA_0000659, hsa_circRNA_0003247, hsa_circRNA_0001535	Hepatotoxicity	-	-
hsa_circ_0072765, hsa_circ_0071410, hsa_circ_0054345	Liver fibrosis	-	- miR-9-5p -
circZFR, circFUT8 circIPO11	Liver cancer	-	-
circMEG3	Liver cancer	inhibits telomerase activity, shortens telomere lifespan, reduces Cbf5	-
circRNA-0067835	Liver fibrosis	promotes cell proliferation, inhibits apoptosis	miR-155 to promote FOXO3a
circ_0091579	Liver cancer	promotes proliferative and metastasis	miR-490-3p
hsa_circ_0003056 hsa_circ_0067127	Carcinoma	-	-
circRNA-1984	HSCs-related to fibrosis	-	miR-146b
circ_0015756	Hepatoblastoma	-	-
hsa_circ_0000594	Hepatoblastoma	-	mir-217/SIRT1 regulatory axis
circFBLIM1	Hepatoblastoma	Promotes cell viability, proliferation, invasion	miR-346-ceRNA to regulate FBLIM1 expression
circHMGCS1	Hepatoblastoma	Regulates proliferation, apoptosis and glutaminolysis	miR-503-5p/IGF/PI3K/AKT axis; regulates IGF2 and IGF1R expression
circ-PWWP2A	Fibrogenesis	Downstream reactor of TGF-ß and LPS	miR-203 and miR-223

http://cgga.org.cn:9091/circRNADisease/ accessed on 17 May 2023. Table 5 demonstrates the upregulated and downregulated circRNAs in the different hepatic diseases while denoting the involved molecular mechanism and/or associated miRNA as retrieved from circRNADisease v2.0 bioinformatics database, http://cgga.org.cn:9091/circRNADisease/, accessed on 17 May 2023. [AKT: AKT serine/threonine kinase; FBLIM1: Filamin-binding LIM protein 1; FOXO3a: Forkhead box O3; IGF: insulin-like growth factor; IGF1R: insulin-like growth factor 1 receptor; JAK2: Janus kinase 2; Keap 1: Kelch-like erythroid cell-derived protein with CNC homology [ECH]-associated protein 1; HSCs: hepatic stellate cells; LPS: Lipopolysaccharide; NAFLD: non-alcoholic fatty liver disease; Nrf2: nuclear factor erythroid 2 [NF-E2]-related factor 2; PI3K: phosphoinositide 3-kinase; PPAR: Peroxisome proliferator-activated receptor; p38MAPK: p38 mitogen-activated protein kinase; SIRT1: Sirtuin 1; STAT5: signal transducer and activator of transcription 5; TGF-ß: transforming growth factor beta].

## Data Availability

All the data obtained and/or analyzed during the current study are available within the manuscript or the bioinformatics databases.

## Supplementary Materials

The following supporting information can be downloaded at: https://www.mdpi.com/article/10.3390/cells13151245/s1, Figure S1: Genomic distribution of human circRNAs; number of circRNAs per chromosome retrieved from circRNADb; Figure S2: CircRNA distribution in different tissues, including the liver; Figure S3: Number of circRNAs in different liver cell lines retrieved from CIRCpedia v2; Table S1: Experiments browsed for circRNAs in liver cancer.
