# Supplementary material for: A Comprehensive Insight and In Silico Analysis of CircRNAs in Hepatocellular Carcinoma: A Step toward ncRNA-Based Precision Medicine
Source: Cells. 2024 Jul 24;13(15):1245. doi: 10.3390/cells13151245 (PMC11312109; doi:10.3390/cells13151245)
Supplement: Supplementary file 1 [file cells-13-01245-s001.zip › cells-3046889-supplementary.pdf]

A Comprehensive Insight and *In-silico* Analysis of CircRNAs in Hepatocellular Carcinoma; a Step-Toward ncRNA-based Precision Medicine

Rana A. Youness [1,*], Hossam A. Hassan[1], Tasneem Abaza[1,2,3], Ahmad A. Hady[4], Hekmat M El Magdoub[5], Mohamed Ali[6,7], Johannes Vogel[8], Markus Thiersch[8], Max Gassmann[8], Nadia M. Hamdy[9,*,#], Mostafa A. Aboouf[8,9,*,#]

In silico database analysis concerning circRNAs (Accessed on May 17, 2023)

GeneCards Version 5.15 (last updated: March 27, 2023)

The human gene database was screened (https://www.genecards.org/) with 415’866 genes, of which 43’617 are HGNC (HUGO Gene Nomenclature Committee) approved, 19’871 disease genes, and 500 Hot genes. There are two categories of genes: 21’667 protein-coding and 292’110 ncRNA genes. Among the latter, there are 120 circRNAs. 100% GeneCards databases that have information about circRNAs are the European Nucleotide Archive (ENA), RNAcentral, DGV, and GeneLoc (undergoing infrastructure transformation).

European Nucleotide Archive (last updated December 1, 2022)

European Bioinformatics Institute (EBI) search was utilized to perform a free text search across ENA data (https://www.ebi.ac.uk/ena/browser/home). Concerning human circRNAs, the search revealed 304 circRNAs with a complete sequence, 8 non-coding, and 73 experiments in 137 studies using 45 human samples.

RNAcentral database v22

Non-coding RNA sequence database from EBI was searched (https://rnacentral.org/). Search terms were RNA AND so_rna_type_name:"Circular_ncRNA" AND TAXONOMY:"9606" AND rna_type:"circRNA" AND has_genomic_coordinates:"True", where 133 human homo-sapiens (has) circRNA, with length 128 to 3626 nucleotides are there.

CircRNAs Bioinformatics Analyses

CircRNADb version 1.0

A human circRNA molecules database containing 32,914 annotated exonic circRNAs. (http://reprod.njmu.edu.cn/cgi-bin/circrnadb/circRNADb.php). Of these, 6608 circRNAs have protein-coding potential, with 72 circRNAs with protein expression evidence and 21 circRNAs with protein-coding potential and expression evidence. However, data browsing by samples/cell type revealed 204 circRNA expressed by HEK293, 277 by neutrophils, 6610 by Hs68, 10697 by H9 hESCs, 3495 circRNA were expressed in leukemia, and 1303 for leukocytes. Normal brain tissue expressed 15266 circRNAs, glioblastoma cells expressed 7445, and oligodendroma expressed 7077 circRNAs. Figure S1

addresses the number of circRNAs per chromosome retrieved from circRNADb.

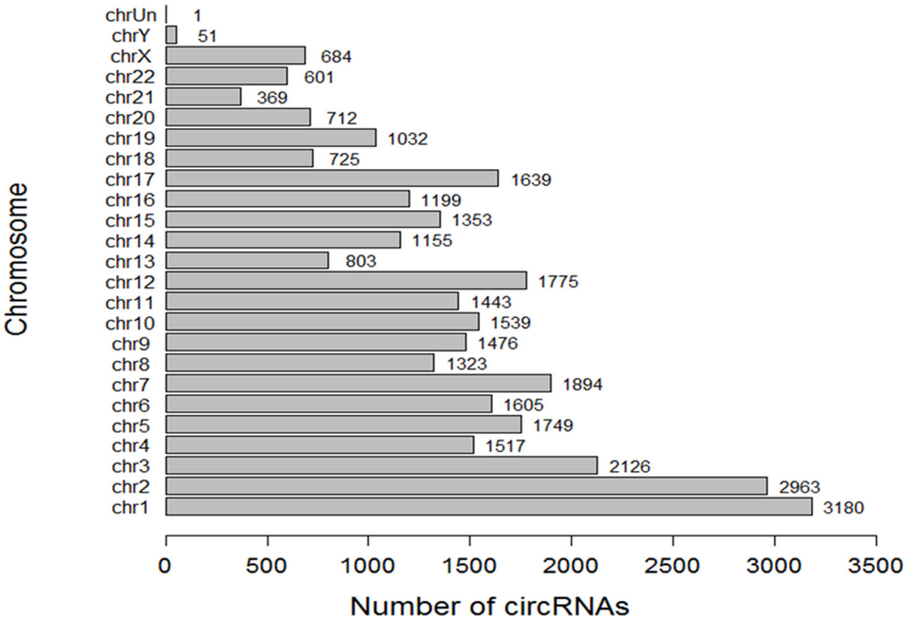

Figure S1: Genomic distribution of human circRNAs; number of circRNAs per chromosome retrieved from circRNADb. It demonstrates the number of circRNAs per chromosome, showing that the largest number of circRNAs are on chromosome #1. http://reprod.njmu.edu.cn/cgi-bin/circrnadb/About_circRNADb.php (accessed on May 17, 2023).

CircAtlas 2.0 (last updated March 30, 2020)

It enables searching the human circRNA by the type of exon, intron, intergenic, antisense, and non-repeat, as well as the 3'-UTR or 5'-UTR, positive or negative strand within different chromosomes (http://circatlas.biols.ac.cn/). Figure S2 shows human circRNA distribution in various body tissues, including the liver, as retrieved by CircAtlas 2.0.

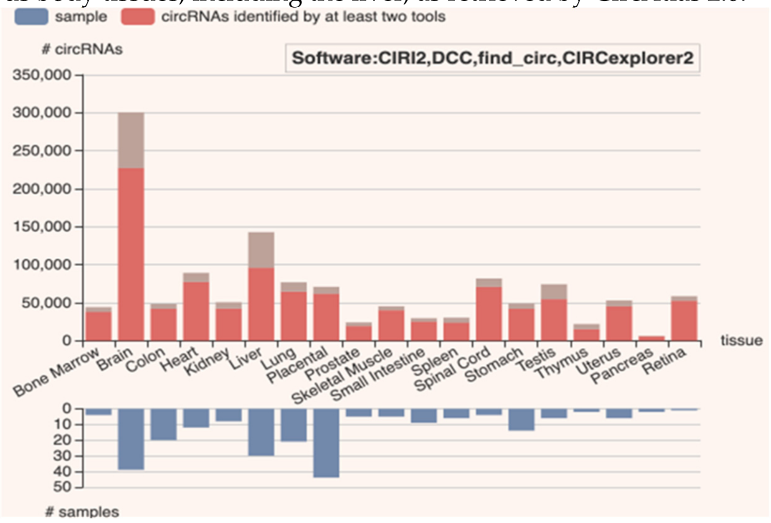

Figure S2: ***CircRNA*** **distribution in different tissues, including the liver** http://circatlas.biols.ac.cn/ It illustrates the distribution of circRNAs in various body tissues, including 30 liver samples (lower inverted panel).

Using CIRCpedia v2 based on the human reference genome, hg19 was added to the CIRCpedia http://yang-laboratory.com/circpedia/ in the liver cell lines, as presented in Figure S3.

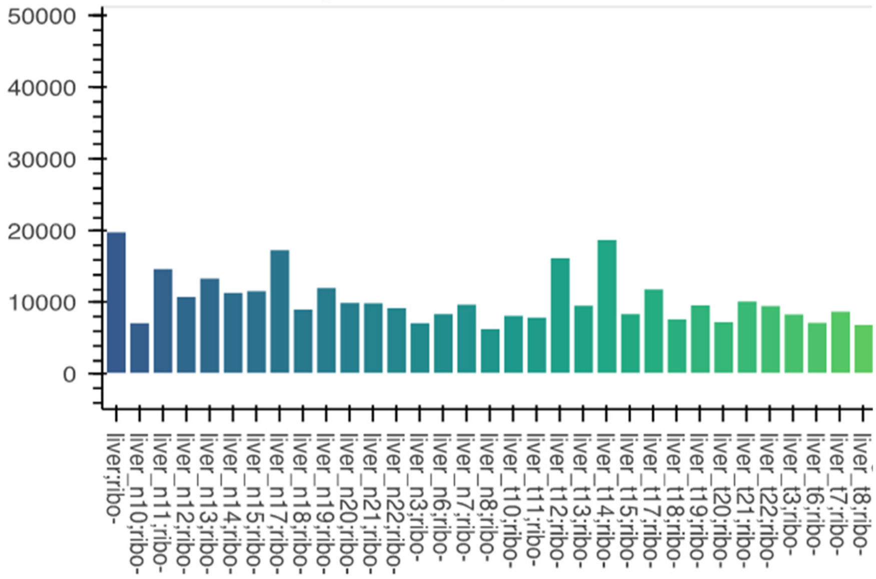

Figure S3: Number of circRNAs in different liver cell lines retrieved from CIRCpedia v2 http://yang-laboratory.com/circpedia/ accessed on May 17, 2023.

CircSC CircRNA in single-cell transcriptomes

Two investigations were conducted using single-cell or combined transcriptomics and functional assessments of HCC cells from 2D and 3D cells, respectively, to assess cancer cell or cancer stem cell heterogeneity. https://ngdc.cncb.ac.cn/circatlas/circSC/data.html accessed on October 10, 2023.

Table S1: Experiments browsed for circRNAs in liver cancer.

Study title	Project ID/ Technology	Description
"Single-cell analysis reveals cancer stem cell heterogeneity in HCC"	GSE103866/ SMART-seq	Combined transcriptomic and functional analyses of HCC cells at single-cell level to assess cancer cell heterogeneity
"Multilineage communication regulates human liver bud development from pluripotency"	GSE96981/ SMARTer	Single-cell transcriptomes, self-organization from pluripotency II

https://ngdc.cncb.ac.cn/circatlas/circSC/data.html accessed on October 10, 2023.
